# Efficacy, quality of life, and acceptability outcomes of atypical antipsychotic augmentation treatment for treatment-resistant depression: protocol for a systematic review and network meta-analysis

**DOI:** 10.1186/2046-4053-3-133

**Published:** 2014-11-05

**Authors:** Yiyun Liu, Xinyu Zhou, Bin Qin, Cinzia Del Giovane, Yuqing Zhang, Peng Xie

**Affiliations:** 1 Department of Neurology, The First Affiliated Hospital of Chongqing Medical University, 1 Yixueyuan Road, Yuzhong District, Chongqing 400016, China; 2 Institute of Neuroscience, Chongqing Medical University, Chongqing, China; 3 Chongqing Key Laboratory of Neurobiology, Chongqing Medical University, Chongqing, China; 4 Department of Diagnostic, Clinical, and Public Health Medicine-Italian Cochrane Centre, University of Modena and Reggio, Emilia Modena, Italy

**Keywords:** Major depressive disorder, MDD, Treatment-resistant depression, TRD, Atypical antipsychotic, Meta-analysis, Systematic review

## Abstract

**Background:**

Major depressive disorder (MDD) is a debilitating and costly mental disorder. Although commercially available antidepressants have proliferated over the last 20 years, a substantial number of patients either do not respond adequately to these drugs or are unable to tolerate their adverse effects. One common approach has been to augment conventional antidepressants with an adjunctive agent, but the optimal selection of atypical antipsychotic agents for adjunctive treatment of treatment-resistant depression (TRD) remains controversial.

**Methods/Design:**

An electronic literature search of PubMed, the Cochrane Library, Embase, Web of Science, LiLACS, CINAHL, and PsycINFO for studies will be conducted with no restrictions on language, publication year, or publication type. Several clinical trial registry agencies, pharmaceutical company websites, and FDA reports will also be reviewed. Randomized clinical trials (RCTs) with atypical antipsychotic augmentation treatment for treatment-resistant depression will be considered. Data will be independently extracted by two reviewers. Traditional pairwise meta-analyses will be performed for RCTs that directly compare different treatment arms. Then, Bayesian network meta-analyses will be performed to compare the relative efficacy and acceptability of different atypical antipsychotic agents (and doses). A sensitivity analysis will be performed by excluding studies classified as a small sample size, having a high placebo effect.

**Discussion:**

This systematic review and network meta-analysis will comparatively analyze the efficacy, quality of life, and acceptability profiles of atypical antipsychotic medications used for the adjunctive treatment of TRD. The findings should provide clinically relevant implications for comprehensively understanding the risk–benefit profiles of these adjunctive treatments.

**Systematic review registration:**

PROSPERO CRD 42014009666.

## Background

Major depressive disorder (MDD) is a debilitating and costly mental disorder. Approximately 5%–12% of males and 9%–26% of females will suffer from at least one episode of MDD over their lifetime, and about 50% of patients will experience a second depressive episode [1-3]. Even though available antidepressants have proliferated over the last 20 years, a substantial number of patients either do not respond adequately to these drugs or are unable to tolerate their adverse effects [4,5]. Recently, the STAR*D have indicated that only approximately half of patients being treated for MDD with antidepressants show a favorable treatment response and only about one third achieve remission [6], highlighting the need for optimized treatment strategies for treatment-resistant depression (TRD).

Atypical antipsychotic medications are widely used in the treatment of MDD. In the United States in 2007 and 2008, there were an estimated 3.7 million treatment visits per year in which an atypical antipsychotic medication was prescribed for depression [7]. Currently, three atypical antipsychotic drugs—aripiprazole, olanzapine, and quetiapine—have received approval from the US Food and Drug Administration (FDA) as adjunctive therapies in adult MDD, while none have been approved as monotherapy [8].

In clinical practice, however, controversy exists as to the optimal selection of a particular atypical antipsychotic medicine for augmentation therapy in TRD patients, as atypical antipsychotics differ in their selectivity for 5-HT receptors and/or D2 receptors as well as their effects on different brain regions [9]. Although the efficacy and tolerability of adjunctive atypical antipsychotic therapy in TRD have been summarized in at least three previous traditional pairwise meta-analyses of randomized control trials (RCTs), none have provided evidence-based hierarchies for the efficacy and tolerability of these atypical antipsychotic drugs [10-12]. More importantly, the question of the superiority of a given dosage in terms of efficacy and tolerability has never been assessed in the comprehensive setting of a systematic review and meta-analysis, as few trials have directly compared different dosages of atypical antipsychotics in MDD [13,14].

To address the foregoing concerns, an approach termed Bayesian network meta-analysis can be applied to integrate direct evidence (from studies directly comparing interventions) with indirect evidence (information about two treatments derived via a common comparator) from multiple treatment comparisons (multiple drugs and multiple doses) to estimate the interrelations across all treatments [15]. This approach enables a coherent analysis of RCT data for comparisons of multiple treatments without adversely affecting randomization of treatments within each trial; the usefulness of this approach has been previously demonstrated in several studies on various medical conditions and interventions [16-19].

### Objectives

In this systematic review and network meta-analysis, we aim to compare the efficacy, quality of life, and acceptability of atypical antipsychotics as augmentation therapy for adults with TRD.

## Methods/Design

### Data sources and search strategy

This systematic review will be reported using PRISMA guidelines [20]. Seven electronic databases (PubMed, Embase, the Cochrane Library, Web of Science, CINAHL, LiLACS, and PsycINFO) and databases of grey literature (System for Information on Grey Literature in Europe [SIGLE] and National Technical Information Service [NTIS]) will be searched from inception up to January 2014 with the following Medical Subject Headings and text words: (*depression* OR *dysthymia* OR *mood disorder* OR *affective disorder*) AND (*atypical antipsychotic* OR *second-generation antipsychotic* OR *aripiprazole* OR *asenapine* OR *clozapine* OR *iloperidone* OR *lurasidone* OR *olanzapine* OR *paliperidone* OR *quetiapine* OR *risperidone* OR *ziprasidone*). Several clinical trial registry agencies, pharmaceutical company websites, and FDA reports will be also reviewed (Additional file 1). There will be no restrictions on language, publication year, or type of publication. Additional studies will be searched in the reference lists of all identified publications including relevant meta-analyses and systematic reviews. All relevant authors and principal manufacturers will be contacted to supplement incomplete reports of the original papers or to provide new data for unpublished studies.

### Study selection

Two reviewers will independently select studies for inclusion with disagreements resolved by consensus. They will scan citations at the title/abstract level and then retrieve short-listed studies in full text. Potentially relevant articles will be reviewed in full length to ensure that they satisfy all inclusion and exclusion criteria as follows:

#### Type of studies

We will include randomized controlled trials that compare an adjunctive atypical antipsychotic medication to another different class (and dosage) of adjunctive atypical antipsychotic medication or placebo.

#### Types of participants

Prospective, consecutive enrollment of adult patients with a primary diagnosis of current unipolar depressive disorder according to standardized diagnostic criteria that displayed an inadequate response to at least one course of antidepressant therapy prior to enrollment in the study (i.e., TRD) is needed. All classes of antidepressants will be included in this study. Studies will be excluded if they included patients with bipolar depression or co-administered a psychotherapeutic intervention.

#### Types of interventions

The intervention of interest is an adjunctive atypical antipsychotic medication compared to another different class (and dosage) of adjunctive atypical antipsychotic medication or placebo. Studies will be excluded if they involve the co-administration of psychotherapy or involve relapse prevention or maintenance treatment. There are no restrictions on classes of antidepressants. A subgroup analysis will be conducted to evaluate the different class of antidepressants (SSRI vs. non-SSRI).

#### Types of outcome measures

Response and remission rates are often used to convey the magnitude of treatment benefit; however, these categorical measures are created arbitrarily from underlying continuous rating scale data [21]. In some circumstances, these categorical measures may inflate treatment differences relative to the mean change on the continuous scale [22]. Therefore, in this study, the primary outcome for efficacy will be chosen as a continuous measure of depressive symptom severity, which will be calculated as the standardized mean difference (SMD) in either the Montgomery-Åsberg Depression Rating Scale (MADRS) [23] or the Hamilton Depression Rating Scale (HAM-D) [24] from baseline to endpoint. A negative SMD value for depression symptoms indicates greater symptomatic relief. When SDs of absolute changes from baseline are not available from individual trials, they will be imputed from *p* values as described in the Cochrane Handbook [25]. In addition, we will assess categorical response rates and remission rates as secondary outcomes for efficacy analysis. Response will be estimated as the proportion of individuals that respond to treatment through a ≥50% decrease in depression rating score from baseline to end point on either the MADRS or HAM-D [26]. Remission will be defined variably across studies. The commonly used definitions of remission include MADRS ≤8, then HAM-D ≤7, then MADRS ≤10 [26]. When data are reported on both the MADRS and HAM-D, we will preferentially use data from the MADRS, as it is the most commonly used measure of depressive symptoms. One or more outcomes of depressive symptoms should be during the acute treatment phase (4 to 12 weeks). For trials with multiple durations of acute treatment, the 8-week outcomes will be used.

We also plan to assess continuous measures of quality of life (QoL), including the Quality of Life Enjoyment and Satisfaction Questionnaire (Q-LES-Q) [27] and the Short Form 36 Health Survey (SF-36) [28]. The only continuous measure of functional impairment that will be employed is the Sheehan Disability Scale (SDS) [29]. As measures of quality of life and functional impairment (QoL/functioning) vary across studies, we will pool such measures together to create an omnibus effect size of SMD for each drug and across all drugs. A negative SMD value for QoL/functioning indicates greater functional improvement. When data are reported on the Q-LES-Q, SF-36, and SDS, we will first choose data from the Q-LES-Q, then SDS, and then SF-36.

The primary outcome for acceptability is all-cause discontinuation, which will be measured as the proportion of patients who dropout for any reason. The secondary outcome for acceptability is side-effects discontinuation, which will be estimated as the proportion of patients who dropout for adverse events. Outcomes will be allocated according to the intention-to-treat principle.

### Data extraction and management

Two independent reviewers will independently extract the key study parameters using a standardized data abstraction form and assess the studies’ methodological quality using the risk of bias assessment tool from the Cochrane Handbook [25]. Disagreements will be resolved by consensus.

### Data collection and analysis

First, traditional pairwise meta-analyses will be performed for studies that directly compare different treatment arms. Then, we will perform Bayesian network meta-analyses to compare the relative efficacy and acceptability of different atypical antipsychotic agents (and doses).

### Traditional pairwise meta-analyses

Traditional pairwise meta-analyses will be performed using Review Manager (version 5.2). Using the DerSimonian method and the Laird random effects model, the pooled estimates of odds ratios (OR) with 95% confidence intervals (CIs) will be calculated for the categorical outcomes and the standardized mean difference (SMD) with 95% CIs will be calculated for the continuous outcomes [25].

### Bayesian network meta-analyses

Network meta-analyses will be performed using the WinBUGS software package (version 1.4.3, MRC Biostatistics Unit, Cambridge, UK) with random effects models for multi-arm trials [15,30]. The pairwise meta-analysis and *I*^2^ calculations will be performed by the Stata 11.0. The estimation of consistency, rankograms, and surface under the cumulative ranking (SUCRA) graphs will be presented by R 2.11.1 software packages. Network meta-analyses will be performed on two different evidence networks. The primary analysis based on a network where different agents (and doses) will be treated as separate nodes with standard adjusted dose aripiprazole (2–20 mg daily, mean 10 mg daily), low dose aripiprazole (2 mg daily), standard adjusted dose olanzapine/fluoxetine (olanzapine 5–20 mg daily, mean 10 mg daily/fluoxetine 25–60 mg daily, mean 40 mg daily), low dose olanzapine/fluoxetine (olanzapine 1 mg daily/fluoxetine 5 mg daily), quetiapine (mean 250–350 mg daily), quetiapine (mean 150–250 mg daily), risperidone (0.25–3 mg daily, mean 1 mg daily), and placebo. A secondary evidence network of drug class will be also constructed to compare the effects of aripiprazole (excluding low dose), olanzapine/fluoxetine (excluding low dose), quetiapine (mean 250–350 mg daily), quetiapine (mean 150–250 mg daily), risperidone, and placebo.

The pooled estimates will be obtained using the Markov Chains Monte Carlo method. Two Markov chains will be run simultaneously with different arbitrarily chosen initial values. To ensure convergence, trace plots and the Brooks-Gelman-Rubin statistic will be assessed [31]. Convergence to a stable solution will be checked by viewing plots of the sampled simulations, and then these samples will be discarded as “burn-in”, and posterior summaries will be based on adequate subsequent simulations [32,33]. All results will be reported as posterior medians of OR or SMD with corresponding 95% credible intervals (CrIs), which can be interpreted like conventional 95% CIs. When a loop connects three treatments, it will be possible to evaluate the inconsistency between direct and indirect evidence. The node splitting method will be used to calculate the inconsistency of the model, which separates evidence on a particular comparison into direct and indirect evidence [34].

The probability of each treatment being the most effective, the second best, the third best, etc., will be calculated and graphically ranked with rankograms [35]. Probability values will be summarized and reported as SUCRA curve, a simple transformation of the mean rank used to provide a hierarchy of the treatments that accounts for both the location and the variance of all relative treatment effects [36]. The larger the SUCRA value, the better the rank of the treatment with a SUCRA of 1.0 if an intervention always ranks first and 0.0 if it always ranks last.

### Assessment of heterogeneity and reporting biases

Heterogeneity of treatment effects across studies will be assessed according pairwise meta-analysis by *I*^2^ and the Cochrane Q test [25]. Publication bias will be examined with the funnel plot method, the Begg’s adjusted rank correlation test, and the Egger’s regression asymmetry test [37,38].

### Sensitivity analysis

In a sensitivity analysis, the network meta-analysis will exclude trials with small sample sizes (i.e., arms of less than 10 patients). Then, another sensitivity analysis will be conducted to examine whether effect estimates are influenced by the placebo effect investigated in the individual trials. Finally, network meta-regression analysis will be used to investigate whether potential heterogeneity can be explained by differences in publication year and the effect of sponsorship.

## Discussion

This review will systematically and comprehensively retrieve a significant amount of published and unpublished evidence from a wide range of sources. Potential bias will be minimized by having a pair of reviewers that independently scan through the search output, extract the data, classify the interventions, and assess the methodological quality of each RCT. The Bayesian random effects model is the most appropriate method for network meta-analysis or mixed treatment comparison. This statistical technique not only includes the results of direct comparisons, but also incorporates indirect comparisons that are rarely included in head-to-head trials, thereby overcoming a major oversight of conventional pairwise meta-analyses. We also specifically plan to investigate side-effects discontinuation, personal or social functioning, and QoL outcomes to more comprehensively assess the adjunctive use of atypical antipsychotic agents. Thus, this systematic review and network meta-analysis will provide useful, hierarchical, and complete evidence on efficacy, QoL, and acceptability of different types (and doses) of atypical antipsychotic medications used for the adjunctive treatment of MDD. This will provide clinically relevant implications for comprehensively understanding the risk–benefit profiles of these adjunctive treatments.

## Abbreviations

TRD: treatment-resistant depression; MDD: major depressive disorder; RCTs: randomized clinical trials; SMD: standardized mean difference; OR: odds ratios; CI: confidence interval; CrIs: credible intervals; HAM-D: Hamilton Depression Rating Scale; QoL: quality of life; MADRS: Montgomery-Åsberg Depression Rating Scale; Q-LES-Q: Quality of Life Enjoyment and Satisfaction Questionnaire; SF-36: Short Form 36 Health Survey.

## Competing interests

The authors declare that they have no competing interests.

## Authors’ contributions

XZ, YL, and PX conceived the idea and designed the search strategy and data abstraction forms. XZ, YL, BQ, CDG, and YZ participated in the design and drafting of the protocol. All authors read and approved the final manuscript.

## Supplementary Material

Additional file 1 **Search strategy.** Search terms for electronic databases. Click here for file
