# Supplementary material for: Efficacy, quality of life, and acceptability outcomes of atypical antipsychotic augmentation treatment for treatment-resistant depression: protocol for a systematic review and network meta-analysis
Source: Syst Rev. 2014 Nov 5;3:133. doi: 10.1186/2046-4053-3-133 (PMC4234739; doi:10.1186/2046-4053-3-133)
Supplement: Additional file 1 — Search strategy. Search terms for electronic databases. [file 2046-4053-3-133-S1.doc]

Results from the Systematic Search Strategy*

| **Databases:** | **Citations** |
| --- | --- |
| Pubmed | 1177 |
| Cochrane | 1010 |
| Web of Science | 1098 |
| Embase | 703 |
| CINAHL | 45 |
| PsycInfo | 812 |
| LILACS | 74 |
| SIGLE | 163 |
| NTIS | 33 |
| ***Total (*databases)** | ***5115*** |
| **Trial registers:** Australian Clinical Trials Registry, USA (clinicaltrials.gov), UN (ISRCTN), Netherlands (Trial Register), Japan (UMIN-CTR), Chinese (ChiCTR), and World Health Organization (WHO) | 340 |
| ***Total*** | ***5455*** |

*Explicit search strategy: title/abstract = (depress* or dysthymi* or “mood disorder*” or “affective disorder*”) AND (atypical antipsychotic OR second-generation antipsychotic OR antipsychotics OR aripiprazole OR asenapine OR clozapine OR iloperidone OR lurasidone OR olanzapine OR paliperidone OR quetiapine OR risperidone OR ziprasidone )

Other sources: Relevant principal manufacturers (e.g., Lilly, AstraZeneca, Bristol-Myers Squibb, Pfizer and Janssen) were contacted. Additional relevant studies were obtained by scanning relevant systematic reviews, meta-analyses, and reviews as well as reference lists of eligible trials.
